# Targeting functional fitness, hearing and health-related quality of life in older adults with hearing loss: Walk, Talk 'n' Listen, study protocol for a pilot randomized controlled trial

**DOI:** 10.1186/s13063-017-1792-z

**Published:** 2017-01-28

**Authors:** Justin Lambert, Rouzbeh Ghadry-Tavi, Kate Knuff, Marc Jutras, Jodi Siever, Paul Mick, Carolyn Roque, Gareth Jones, Jonathan Little, Harry Miller, Colin Van Bergen, Donna Kurtz, Mary Ann Murphy, Charlotte Ann Jones

**Affiliations:** 1 Faculty of Medicine, Southern Medical Program, University of British Columbia, Okanagan campus, Kelowna, BC Canada; 2 Faculty of Medicine, Department of Surgery, Division of Otolaryngology, University of British Columbia, Okanagan campus, Kelowna, BC Canada; 3 Faculty of Health and Social Development, School of Health and Exercise Science, University of British Columbia, Okanagan campus, Kelowna, BC Canada; 4 Irving K. Barber School of Arts and Social Sciences, Psychology, University of British Columbia, Okanagan campus, Kelowna, BC Canada; 5 Audiologist, NexGen Hearing, Kelowna, BC Canada; 6 Faculty of Health and Social Development, School of Nursing, University of British Columbia, Okanagan campus, Kelowna, BC Canada; 7 Irving K. Barber School of Arts and Social Sciences, Sociology and School of Social Work, University of British Columbia, Okanagan campus, Kelowna, BC Canada; 8 Southern Medical Program, #321 Reichwald Health Sciences Center, 1088 Discovery Ave, Kelowna, BC V1V-1V7 Canada

**Keywords:** Hearing loss, Disability, Functional fitness, Auditory rehabilitation, Loneliness, Socialization, Older adults

## Abstract

**Background:**

Hearing loss (HL) is a disability associated with poorer health-related quality of life including an increased risk for loneliness, isolation, functional fitness declines, falls, hospitalization and premature mortality. The purpose of this pilot trial is to determine the feasibility and acceptability of a novel intervention to reduce loneliness, improve functional fitness, social connectedness, hearing and health-related quality of life in older adults with HL.

**Methods:**

This 10-week, single-blind, pilot randomized control trial (RCT) will include a convenience sample of ambulatory adults aged 65 years or older with self-reported HL. Following baseline assessments, participants will be randomized to either intervention (exercise, health education, socialization and group auditory rehabilitation (GAR)) or control (GAR only) groups. The intervention group will attend a local YMCA twice a week and the control group once a week. Intervention sessions will include 45 min of strengthening, balance and resistance exercises, 30 min of group walking at a self-selected pace and 60 min of interactive health education or GAR. The control group will attend 60-min GAR sessions. GAR sessions will include education about hearing, hearing technologies, enhancing communication skills, and psychosocial support. Pre-post trial data collection and measures will include: functional fitness (gait speed, 30-s Sit to Stand Test), hearing and health-related quality of life, loneliness, depression, social participation and social support. At trial end, feasibility (recruitment, randomization, retention, acceptability) and GAR will be evaluated.

**Discussion:**

Despite evidence suggesting that HL is associated with declines in functional fitness, there are no studies aimed at addressing functional fitness declines associated with the disability of HL. This pilot trial will provide knowledge about the physical, mental and social impacts on health related to HL as a disability. This will inform the feasibility of a larger RCT and preliminary evidence about the initial effects of a novel, community-based, holistic intervention addressing both the negative psychosocial and functional physical effects of HL among older adults.

**Trial registration:**

ClinicalTrials.gov, NCT02662192. Registered on 14 January 2016

**Electronic supplementary material:**

The online version of this article (doi:10.1186/s13063-017-1792-z) contains supplementary material, which is available to authorized users.

## Background

### Conceptual framework

**Fig. 1:**
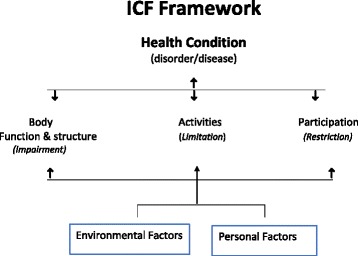
The World Health Organization International Classification of Functioning, Disability and Health framework

The World Health Organization (WHO) International Classification of Functioning, Disability and Health (ICF) [1] is a clinically relevant framework that describes disability in terms of physical impairments, activity limitations, participation restrictions and the contextual factors (environmental and personal) that interact to influence the disability (Fig. 1). The ICF provides an excellent, inclusive framework to consider the far-reaching effects of hearing loss (HL) as a disability and to evaluate the influence of interventions, such as the Walk, Talk ‘n’ Listen (WTListen) program, on participants with HL.

Hearing loss is one of the most common chronic health problems in North America. Analysis of the audiometry data from the 2001–2008 cycles of the National Health and Nutrition Examination Surveys revealed that nearly 20% of Americans of 12 years of age or older have unilateral or bilateral HL [2]. The prevalence of HL increases with age. Audiometry results from the 2012 and 2015 Canadian Health Measures Survey [3] indicated that 78% of adults aged 60 to 79 years have audiometrically measured HL and that over 77% are undiagnosed.

The disability of HL may influence or be influenced by interactions between all domains of the ICF including personal factors (e.g., socioeconomic, education, coping mechanisms), limitations to environmental factors (e.g., relationships, supports, attitudes), body functions (e.g., declining auditory, cognitive and musculoskeletal function), limitations in activities of daily living (e.g., self-care, mobility), and participation restrictions (e.g., communication, relationships, social life). Epidemiologic studies have established independent associations between HL and poorer health-related quality of life (HRQOL: physical and mental health domains) [4, 5], social isolation [6, 7], depression [8], incident dementia [9], cognitive decline [10], increased physical dependence in activities of daily living (admission to a nursing home or requiring assistance at home) [7, 11], increased sedentary time [12], decline in functional fitness [13] including gait speed [14, 15], increased falls [16], hospitalizations [17] and a near 36% increase in age- and sex-adjusted cardiovascular and all-cause mortality (ACM) [18–21]. Applying the ICF framework to those with HL suggests that the ideal intervention aimed at addressing HL as a disability should be holistic and address personal, environmental, psychosocial and physical domains.

Provision of hearing aids (HAs) and one-on-one or group auditory rehabilitation (GAR) are currently the most common approach to treating HL. Providing HAs, education about hearing and hearing devices/technologies, psychosocial support and enhancing communication skills are the primary components of auditory rehabilitation [22]. Effective one-on-one or group auditory rehabilitation (AR) optimizes environmental and personal functioning and with the provision of HA, may address body functions (impaired auditory function) along with decreasing activity and participation limitations [23–26]; however, it does not address the well-documented declines in functional fitness (musculoskeletal: gait speed, activates of daily living (ADL) performance and increased risk for falls).

While there is some longitudinal observational evidence that two or more sessions of “muscle strengthening” exercise per week may increase longevity among adults with moderate to severe HL [27], there are no published interventions addressing the ICF domains of body function, activity and participation limitations related to declines in functional fitness in older adults with HL. Consistent with the ICF approach to HL as a disability, Chia et al. [5] emphasize the importance of understanding the synergist effects of physical disabilities, medical and social conditions on HRQOL in older adults. There is a need for more research exploring the effectiveness of strategies that not only address the ICF domains of activity and participation limitations related to impaired auditory function but that also improve functional fitness, gait speed and ADL, all of which are negatively impacted by HL.

Based on the literature and cocreated in collaboration with nearly 400 low-income seniors, Walk ‘n’ Talk for Your Life (WTL) [28] is a 10-week community-based program of socialization/health education (SHE) and graduated physical exercise program for older adults at risk of social isolation and loneliness (SI&L). While not designed for participants with HL, 30 of the first 150 WTL participants self-reported HL [29]. Compared to those without HL, participants with HL tended at baseline to be lonelier, to have a higher prevalence of possible mild depression and to have lower levels of functional fitness. By program end, both those with and without HL showed promising improvements in functional fitness and decreases in loneliness measures. In-depth, one-on-one interviews were performed with participants with self-reported HL to answer two questions: (1) Did HL affect the acceptability of WTL and if so, how? and (2) What did they feel might improve the program to address the impact (if any) of HL? From this information, (manuscript submitted) the WTListen intervention tailored for older adults with HL was developed.

In partnership with the Young Men’s Christian Association (YMCA) of Okanagan, the aim of this pilot randomized controlled trial (RCT) is two-fold: (1) to explore the feasibility and acceptability of the novel WTListen intervention for older adults with HL and (2) to provide preliminary information about the research question: In older adults with HL, what effect does a group exercise and socialization/health education intervention added to GAR have on: (a) body function impairments: (functional fitness) and activity limitations and participation restrictions (hearing-related quality of life, HRQOL) and (b) perceptions of loneliness and social network?

## Methods

### Trial design

**Fig. 2:**
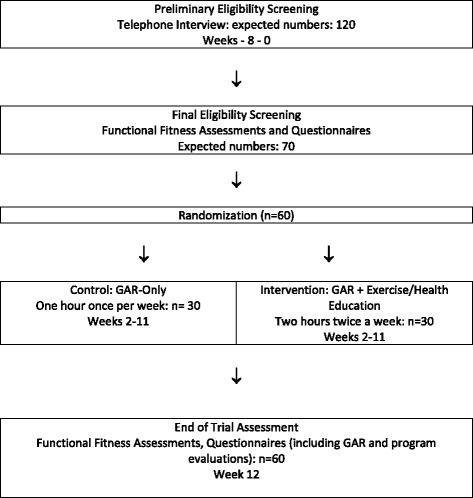
Participant time line: Consolidated Standards of Reporting Trials (CONSORT)-style flow chart

**Fig. 3:**
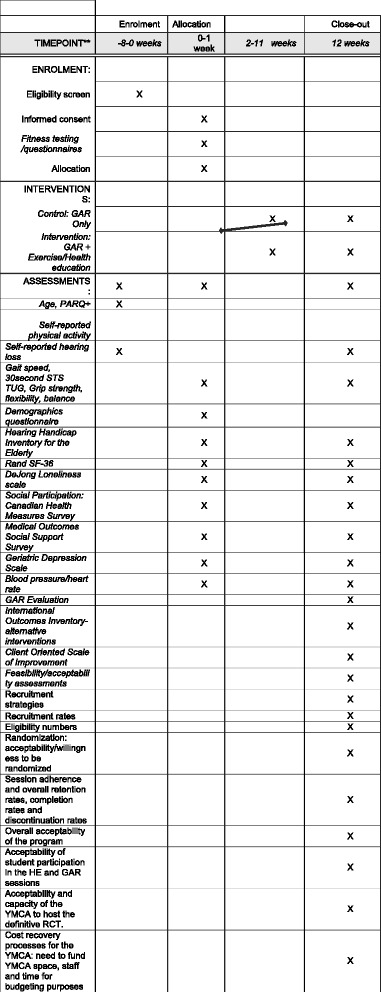
Standard Protocol Items: Recommendations for Interventional Trials (SPIRIT) figure. *Recommended content can be displayed using various schematic formats. See SPIRIT 2013 Explanation and Elaboration for examples from protocols. **List specific timepoints in this row

In this single-blind, randomized controlled pilot trial, 60 ambulatory adults aged 65 years or older, with self-reported HL will be randomized into either the WListen intervention group (exercise, SHE sessions and GAR) or the control group (GAR alone) (see Fig. 2 and the Standard Protocol Items: Recommendations for Interventional Trials (SPIRIT) Checklist (Additional file 1) and the SPIRIT figure (Fig. 3)). Control-group participants will be asked not to change their current physical activity levels and will be offered the 10-week exercise component after the trial is complete. The trial will take place in the “real life” context of a local sports and recreation facility (YMCA Okanagan) and will be free of charge to all participants. Interactive GAR and SHE sessions will be small, closed groups of no more than 12 participants and facilitated for the most part by CAJ and KK. An audiologist (KVB) will deliver a GAR session on the anatomy and process of HL and hearing assistive technologies. This trial will examine recruitment efficacy, reasons for participant interest in joining the trial, attrition rates and reasons, acceptability of GAR, SHE and physical activity interventions along with changes in the functional fitness and psychosocial measures relative to the control group. The findings will inform the design of a larger, multisite RCT.

#### Trial population and randomization

Ambulatory, community-dwelling adults aged 65 years or older will be invited to participate either directly by their audiologist, or through posters and information sheets placed in 10 Kelowna audiologists’ offices, otolaryngologists’ offices, local seniors’ venues, the YMCA, local nonprofit seniors’ agency newsletters and local newspaper ads. Potential participants who call the trial telephone number will be given information by the trial coordinator (TC: CR) about the trial and, if still interested, will undergo a preliminary telephone eligibility assessment.

#### Preliminary telephone eligibility assessment

Table 1 provides the detailed inclusion and exclusion criteria. The trial coordinator, the principle investigator (PI: CAJ), will verbally review the Consent Form with potential participants and answer any questions that arise.. After verbal consent is obtained, participants will be again asked, “Do you have difficulty hearing when conversing with another person in a noisy environment?” [30]. Those answering “yes” will be guided through the validated Physical Activity Readiness Questionnaire (PARQ+) [31] to confirm that they meet the inclusion criteria and are healthy enough to participate in the intervention without exacerbating any existing symptomatology [32]. Those who pass the initial PARQ+ screen and/or those who provide a physician-signed CSEP letter of “exercise readiness” and:

**Table 1:** Inclusion and exclusion criteria of the Walk, Talk ‘n’ Listen trial

Inclusion criteria	Exclusion criteria
1. Aged 65 years or older 2. English speaking 3. Able to sign written informed consent 4. Hearing Handicap in the Elderly (HHIE-25) score of >17 [47] or a previous diagnosis of HL 5. Clearance to safely partake in the trial’s physical activities: PARQ+ (Physical Activity Readiness Questionnaire [66]) or written physician clearance for participation 6. Moderate functional fitness: gait speed >0.72 and <1.8 m/s [67] and/or 30-s STS ≥6 and <21 (within or below published sex- and age-adjusted average levels)	1. Unable to ambulate/walk for exercise 2.Serious illness limiting their ability to exercise or complete the trial 3. Contraindications to exercise: failure to fulfill the prerequisites of the PARQ+ 4. Uncontrolled hypertension (≥160/>90 mmHg) 5. Signs or symptoms of alcohol or substance dependence 6. Refusal to be randomized 7. Lack of transportation to the trial 8. Unable to commit to attending 80% or more of the sessions

*30-s STS* 30-s Sit to Stand Test, *HL* hearing loss

- Self-report less than 150 min per week of physical activity [33]
- Have not participated in any organized exercise program for at least 6 months
- Are available and willing to attend at least 80% of the 10-week sessions in addition to completing baseline and final assessments, will be invited for final eligibility assessment

#### Final eligibility assessment

Final eligibility assessment (functional fitness testing) and baseline questionnaire completion will take place at the trial site (a local YMCA site) and be performed by members of the trained research team after signed informed consent has been obtained.

#### Randomization

Participants will be randomized (Stata® (StataCorp. 2013. Stata Statistical Software: Release 13, College Station, TX, USA: StataCorp LP) by an independent statistician (JS) into two groups, using permuted blocks of random sizes, stratifying on gender and age (below 73 years/73+ years) to ensure even distribution of these variables. The block sizes will not be disclosed to ensure concealment [34, 35]. When either of a couple is randomized to a different group (one to the control group and one to the intervention group), a fair coin toss will be used to decide which group they will both be assigned to (heads = control; tails = intervention). The randomization sequence will be concealed from the researchers who will confirm consent and eligibility with participants before allocation is revealed. Participants will be enrolled and assigned to the time and day(s) of the week by the project coordinator in consideration of their personal schedules. It is not feasible to mask participants or researchers after group allocation as the intervention includes an exercise program and the control does not. 

### Research team development

The research team will include the PI, the PC, two to four students per semester and their faculty supervisors (CAJ, HM, DK, GJ, JL, M-AM) from medicine, psychology, nursing, human kinetics and social work, respectively. This research team will be responsible for pre-and post-intervention assessments and training students and staff to conduct sessions in either the intervention or the control group.

### Intervention

The trial will occur at different times on the same days for the intervention and the control groups. The intervention groups (GAR-SHE-exercise) will visit the YMCA twice a week (2 days apart) for 10 weeks. On the first visit each week they will attend a 1-h interactive GAR session (details of the session will be the same as for the control groups) and 90 min of exercise and walking. On their second visit each week they will attend a 1-h interactive SHE session followed by 90 min of exercise and walking. The exercise intervention will be offered to the control group after the RCT is completed (weeks 13–24). Control groups (GAR only) will attend a 1-h interactive GAR session at the YMCA once a week for 10 weeks. Trained students will help the PI to facilitate the GAR and SHE sessions. GAR sessions will be held in the same small, carpeted room and exercise sessions will occur in a small gym facilitated by a fitness instructor using a microphone and FM amplification system.

#### GAR sessions

GAR sessions (control and intervention groups) will be guided by a modification of the GROUP program [36] (http://idainstitute.com/toolbox/group/). The GROUP program is an IDA Institute-sponsored, web-based, interactive video-enabled program that provides a step-by-step guide for implementing and facilitating GAR programs. The guide provides instructions and informational content/handouts of best practices informed by leading GAR experts along with ethnographic videos allowing facilitators to see GAR in action. In addition, an audiologist (CVB) will facilitate the GAR session on HAs and hearing assistive technology for all groups. GAR sessions will include ice-breaking activities, ground rules for participants, goal setting, multiple communication strategies, coping with HL, handling difficult listening situations, types and uses of hearing assistive technologies, local resources and “advocating for yourself and others with HL.” Psychosocial, mindfulness and stress-reduction strategies will also be included. Participants will engage in practical exercises to do as a group and at home. They will be encouraged to review their GAR handouts with their communication partners (CPs: spouse, significant other or friend). In addition to the weekly sessions, a single 3-h session will be scheduled to include participants’ CPs. In this session, participants and their communication partners will discuss their own communication challenges and together decide upon and practice relevant communication strategies.

#### Socialization/health education (SHE) sessions

The interactive SHE sessions (intervention group only) will begin with a physical activity goal-setting session while the subject matter of the remaining nine SHE sessions topic areas will be decided upon by trial participant consensus. As for the WTL program [28], these sessions will, for the most part, be developed and facilitated by the students although some invited speakers will facilitate sessions in their area of expertise.

#### Exercise and walking sessions

A certified YMCA trainer will facilitate the 1-h exercise and 30-min walking-track sessions. These sessions will follow the standardized YMCA Fit for Life 50+ Program (https://www.h2okelowna.ca/Programs/Health-Fitness/Land-Fitness/Fit-for-Life-50?location=13ee95d3-cc67-48ca-9adb-c05d2d27fdc4) designed to build up strength, movement, coordination and balance. It incorporates TRX™, free weights and the walking track. Participants who miss an exercise session are asked to “make each one up” by either attending another Fit for Life 50+ Program session or doing a set of home-based Otago Falls Prevention Program exercises [37]. Participants are also encouraged to walk between trial sessions and will be provided a pedometer and tracking sheets to motivate and encourage them.

The interactive GAR and SHE sessions will begin with structured goal-setting interviews based on the model of social cognitive theory of behavior change [38], motivational interviewing [39] and collaborative goal setting [40]. Two to three specific, measurable, achievable, realistic goals for both auditory and physical activity outcomes will be identified and prioritized by participants. Goal setting and attainment will be revisited at each session using the social cognitive approach to motivate, empower and encourage adherence.

### Measures

The primary measures: feasibility outcomes and acceptability of the pilot RCT:

1. Recruitment strategies (how did participants hear of the trial, willingness of hearing clinics to recruit participants, number of potential participants contacting the research team and by consulting the pilot trial participants, optimal ways to reach out to isolated individuals with HL)
2. Recruitment rates: numbers of potential participants that contact the trial center; of those, how many participated in telephone interview, how many gave verbal consent, and completed functional physical fitness testing and baseline questionnaires
3. Eligibility: how many potential participants were eligible, how many injuries, adverse events or dropouts)
4. Randomization: acceptability/willingness to be randomized, how baseline measures compared between groups
5. Session adherence and overall retention rates (intervention versus control groups’ daily sign in sheets), final questionnaire completion rates and discontinuation rates (and reasons if given)
6. Overall acceptability of the program (control versus intervention) and GAR, SHE and exercise components (participant evaluation questionnaire: Likert-style and open-ended questions)
  What aspects need to change? What should those changes be and how?
  Acceptability of student participation in the HE and GAR sessions; capacity for student trainees – benefit to research and community? Role or impact of older adult/student relationship might be something to measure in relation to loneliness…
  Acceptability and capacity of the YMCA to host the definitive RCT
  Cost recovery processes for the YMCA: need to fund YMCA space, staff and time for budgeting purposes

The secondary measures: participant-specific outcomes (defined below) in order to generate estimates of data variation (standard deviations (SDs), standard error of the means (SE)), 95% confidence intervals (CIs) around the differences between control and intervention groups, and to determine the sample size estimate for the primary outcome of the definitive RCT:

1. Questionnaire measures:
  data collected at initial assessment include: age, sex, living situation (alone or with someone), marital status, ethnicity, highest level of education, annual household income before taxes, employment status, use of mobility or balance aids, falls over the previous 3 months, and HA use
2. Functional fitness measures:
  measures taken at initial assessment and at the end of the 10-week intervention will include a battery of tests found to be reasonable estimates of the level of fitness associated with remaining physically mobile and independent in later life [41]. All assessments will be conducted over the 1-week period immediately before and at the end of the trial at the same locale using the same protocol and instruments. With the exception of the 6-min Walk Test (6MWT), all tests will be repeated twice for each limb (as appropriate) and the better of the two measures will be recorded (for each limb as appropriate).
  Muscular endurance of the lower limbs will be assessed using the 30-s Chair Stand Test (30SCST) [42]
  Aerobic fitness using gait speed in a 6MWT [43]
  Agility and balance using the Timed Up and Go Test (TUGT) [43]
  Grip strength (isometric muscular strength of the hand and forearm) [44] using a Smedley handgrip dynamometer (Fabrication Enterprises, Elmsford, NY, USA)
  The One-foot Balance Test [45] to examine balance and leg strength/endurance
  Flexibility (lower limbs and lumbar spine) using the Chair Sit and Reach Test [46]; the Back Scratch Test to assess the general shoulder range of motion [41]
3. Measures of hearing and health-related quality of life: (ICF outcomes: activities limitations, participation restrictions) at initial and end of intervention will include:
  The Hearing Handicap Inventory for the Elderly (HHIE-25) [47], a validated 25-item questionnaire assessing the social, emotional and psychological challenges associated with HL and correlates well with audiometrically measured moderate to severe HL
  The RAND SF-36 [48] (ICF outcomes: physical function, activities limitations, participation restrictions, a 36-item health-related quality of life measure with eight subscales including physical functioning, role functioning, bodily pain, general health, vitality, mental health, emotional role limitation and social functioning and social support
4. Measures of loneliness and social connectedness at initial and end of intervention:
  De Jong-Gierveld Loneliness Scale [49])
  Social participation using eight items developed for the Canadian Community Health Survey 4.2 [50], to determine the frequency of participation in family, friendship, and activities with other people outside of the household
  Availability of social support using the Medical Outcomes Trial-Social Support Survey [51], a validated scale of overall social support and four domains of social support (emotional/informational, tangible, affectionate and positive interactions)
  The Geriatric Depression Scale, a15-item questionnaire used as a screening tool in the older population [52]
5. Blood pressure and heart rate (initially and at end of intervention) according to Canadian Hypertension Education Program guidelines [53] using the validated BPM-100 (BpTRU Medical Devices, Coquitlam, BC, Canada), an automated oscillometric noninvasive blood pressure monitor
  Measures taken at the end of the trial.
6. GAR evaluation: at end of intervention. The International Outcomes Inventory-Alternative Interventions (IOI-AI) [54] questionnaire to determine outcomes of GAR programs. A modified Client Oriented Scale of Improvement (COSI) questionnaire [26] to evaluate the extent to which individual goals were reached [55] and overall benefit of the GAR intervention

### Serious adverse events

The trial is expected to be low risk for serious adverse events, such as cardiovascular events (myocardial infarction, stroke, etc.), given the validated PARQ+ screen and/or the provision of a physician signed letter of “exercise readiness.” While the risk is low, there is a possibility of a fall and or fracture during the supervised exercise sessions. This risk will be minimized with exercise sessions facilitated by Canadian Society for Exercise Physiology (CSEP) certified fitness trainers. If an adverse event does occur, the PI (clinical team member onsite during all session times) and key YMCA staff will be immediately alerted and, research protocols and institute appropriate procedures initiated and changes to the exercise program implemented if deemed necessary.

#### Sample size

The sample size for this pilot trial [56, 57] is based upon anticipated numbers of potential participants who contact the trial center within an 8-week recruitment period. Based on previous unpublished experience in the WTL program using pre-post data on older adults with HL, we estimate that approximately 15 per week will contact the trial center, 50–60% of those who make initial contact will meet the eligibility criteria and agree to be randomized, and at least 23 people per group at trial end to show a clinically meaningful average increase in the Sit to Stand Test (STS) of 2 [58]. This sample size will also ensure that enough data is available to generate reliable SE, SD and 95% CI on the sample size required for the large RCT with this measure as the primary outcome. A definitive RCT will be deemed feasible when at least 120 individuals contact the pilot trial center, ≥90% fulfill feasibility outcomes 2–4 and at least 70% of randomized participants fulfill outcome number 5. A larger RCT will be deemed acceptable if at least 85% of participants find the GAR, exercise and SHE sessions highly acceptable or acceptable.

#### Research data and management

Participants will be assigned a participant number upon initial contact with the trial coordinator. Questionnaire and functional fitness testing data will be collected and recorded by the trained research team members on paper-based data collection sheets during the week prior to randomization and during the week after the end of the 10-week trial. Fully anonymized data will be manually entered into an Excel® spreadsheet, 100% double-checked for errors or omissions by a team member blinded to the participants’ group allocation, then cleaned and transferred into Stata® statistical software for analysis.

#### Statistical methods

For primary outcome measures, analyses will be descriptive and variables will be expressed as frequency and percentage for all data relating to recruitment, adherence, overall retention rates, plus all other categorical data on program feasibility and acceptability. Any continuous data will be expressed as mean plus SD or median and interquartile range (for non-normal data). Participant demographics at baseline will be described both by group and overall sample. Responses for Likert-type data will be combined into three nominal categories (“strongly agree/agree,” “strongly disagree/disagree” and “don’t know”) and differences between the intervention and control groups analyzed by Fisher’s exact test [59]. Responses to open-ended questions will be coded and organized into themes and descriptive statistics (including percentages) will be used to report the results.

For secondary outcomes measures, the main analysis will be intention-to-treat: the group to which a participant is assigned will be the group in which they are analyzed, regardless of participant protocol violations, attendance rate or dropout [60]. Last observation carried forward will be used to impute missing outcome data assuming less than 20% missing data for a given outcome measure. The functional fitness measures will be analyzed using the analysis of covariance method with the baseline measure as the covariate and follow-up measure as the outcome [61]. Data will be transformed for analysis of covariance when initial and end of intervention data is non-normal. Both a complete case and per protocol analysis will also be conducted to study the impact of departures from the assumptions made in the main intention-to-treat analysis. All continuous primary and secondary outcome variables will be assessed for normality visually using histograms and boxplots, with the Shapiro-Wilk test used as a supplement to the graphical assessment.

### Knowledge translation

Overall knowledge translation goals will be to increase public and academic awareness of HL as a disability and the need for organized screening initiatives and enhanced programing to support all five ICF domains of disability in older adults with HL. Results will be presented to participants, families and significant others/supports, study partners, at public forums, at local, national and international university academic and health conferences, to health and non-health-related governmental departments and media (radio, local TV). Articles will be published in local newspapers and peer-reviewed academic journals.

## Discussion

HL affects well over half of older adults in Canada and is under-recognized, undertreated and associated with psychosocial and cognitive decline, increased falls, hospitalizations and premature mortality. If successful, dissemination of this unique program may ultimately be associated with significant improvements in the health and wellbeing of older adults with HL. The trial will help to determine the feasibility and acceptability of this program and provides a participatory approach to a detailed intervention plan and sample size considerations for a larger validation RCT. The aim of this pilot trial is to better understand how to access and recruit older adults with HL and how to improve their hearing and health-related quality of life. The unique aspect of this trial is the potential for this intervention to address all five ICF domains of disability by providing access to the environment (YMCA), to support personal factors (information, communication strategies, socialization, goal setting, motivational peer support) that are the foundations for improvements in the physical and psychosocial aspects of activity limitations and participation restrictions. Specifically, the trial will help to understand the potential of this program to improve functional fitness and the psychosocial declines associated with HL.

The functional fitness outcome measures were chosen specifically because of their validated [41] relationship to the maintenance of physical independence for older adults: one of the future large RCT’s main goals. Because of the well-recognized challenges in evaluating GAR outcomes [62], multiple assessment tools were used. For example, the HHIE-25 may not be a sensitive tool to assess some of the emotional/psychosocial changes that a generic quality of life/health scale such as the RAND SF-36 might be. On the other hand, the RAND SF-36 does very little to assess HL-related issues [62]. Use of the HHIE-25, COSI and IOI-A may provide a more global assessment. Furthermore, because one or more of these scales is commonly used in other studies, our data will likely be more readily comparable the other studies. Measurement of loneliness, isolation and social connectedness are also fraught with challenges. It is hypothesized that use of validated scales, especially ones with an extended track record of use in older adult populations [49] or linked to longitudinal health measures surveys, such as the Canadian Community Health Survey, will provide a reasonably valid assessment of change.

Walk, Talk ‘n’ Listen will use a modification of the Otago exercise program that has been associated with 30–35% reductions in falls and fall-related injuries and hospitalizations in the elderly [37]. Along with addressing risk factors for falls, WTListen emphasizes healthy lifestyles, socialization, empowerment in addition to optimizing the hearing and communication of participants through GAR. As such, WTListen has the potential for significant improvements in quality of life, functional fitness and health and in health care dollar savings. Partnering with the YMCA and interdisciplinary student involvement helps to keep expenses down, provides for a rich interdisciplinary community service learning and leadership experience, provides both participants and students with a unique intergenerational experience and provides a vehicle to sustain the program (the WTL program is currently being incorporated into the community service learning curriculum of the UBC Southern Medical Program and integrated with ongoing YMCA programing). While there is research on the disability incurred by those with HL and their family members, there is a paucity of interventions addressing the functional physical fitness ICF domain. This may be an important new area of research into overcoming some of the barriers to a complete and satisfying life in older adults with HL.

There are limitations to this pilot trial. It is not known how readily participants will step forward to participate in the trial. Aside from the well-known reasons for declining participation in research studies, such as lack of time, mobility challenges, lack of awareness of the trial and the desire not to be randomized into a control group, we expect that the presence of HL itself to be a barrier to participation. Older adults experiencing HL may be reluctant to participate in group activities by nature of the effect of their HL on activity and participation limitations especially in group situations. In addition, there is evidence of a link between specific attitudinal beliefs and help-seeking behavior in adults with HL [63] such as perceived lack of severity of their HL, perceived lack of benefit from the clinical trial and perceived lack of self-efficacy. Furthermore, while participants are not obligated to provide reasons for nonadherence or declining participation, we will attempt to secure this information while adhering to standard ethical guidelines. Due to unforeseen circumstances, some of the team members collecting functional fitness data at the end of the trial may not be blinded as to the participants’ allocation to control or intervention group. This will be mitigated in part by assuring that those team members are not assigned to the primary outcome fitness assessments. Additionally, since the PI (CAJ) will be delivering the GAR and SHE sessions and will not be blinded to the participants’ group allocation, she will not perform any primary outcome fitness assessments. Blinding of outcome assessments will be of primary importance in the larger RCT. While the YMCA is highly accessible, transportation challenges may be an issue for some potential participants. Despite the fact that older adult peers with HL essentially designed the program, there may be other unrecognized factors (that we hope to uncover) that may preclude participation.

Strengths of the pilot trial design include the fact that the protocol was developed collaboratively with older adults who have HL. Thus, it is likely to be more suited to the needs and comfort level of those with HL and, therefore, be more acceptable to them.

This pilot project will generate information about the feasibility, acceptability and the implementation of a novel community-based group intervention aimed at reducing the downstream effects of HL among older adults. This knowledge will help to increase awareness of the plight of older adults with HL. This innovative and timely trial will be the first to provide early evidence for the possible benefits of combining socialization, health education and functional fitness training as an approach to addressing major health care gaps in the holistic management of HL [64] and will help to inform necessary changes in health care screening, practice and policy.

### Trial status

Recruitment started in March 2016 and is ongoing until 26 September 2016.

## Additional file

Additional file 1: CONSORT checklist for pilot trials. (DOCX 46 kb)

## Abbreviations

AR: Audiological rehabilitation
CP: Communication partner
GAR: Group audiological rehabilitation
HA: Hearing aid
HE: Health education
HHIE: Hearing Handicap Inventory for the Elderly
HL: Hearing loss
HRQOL: Health-related quality of life
ICF: International Classification of Functioning, Disability and Health
UBCO: University of British Columbia, Okanagan campus, Kelowna, BC, Canada
WHO: World Health Organization
WTL: Walk and Talk for Your Life
WTListen: Walk, Talk ‘n’ Listen
